# A 3-Year Observational Study of Patients with Progressive Systemic Sclerosis Treated with an Intensified B Lymphocyte Depletion Protocol: Clinical and Immunological Response

**DOI:** 10.3390/jcm10020292

**Published:** 2021-01-14

**Authors:** Daniela Rossi, Savino Sciascia, Irene Cecchi, Marta Saracco, Erika Montabone, Vittorio Modena, Raffaele Pellerito, Renato Carignola, Dario Roccatello

**Affiliations:** 1 Center of Research of Rheumatology, Nephrology and Rare Diseases, Coordinating Center of the Interregional Network of Rare Diseases of Piedmont and Aosta Valley, SCDU CMID-Nephrology and Dialysis G. Bosco Hospital and University of Turin, 10154 Turin, Italy; daniela.rossi@unito.it (D.R.); savino.sciascia@unito.it (S.S.); irene.cecchi@unito.it (I.C.); modenavittorio@libero.it (V.M.); 2 Rheumatology Unit, Mauriziano Umberto I Hospital, 10128 Turin, Italy; marta.saracco@gmail.com (M.S.); raffaele.pellerito@gmail.com (R.P.); 3 Internal Medicine, San Luigi Gonzaga Hospital Orbassano, 10043 Turin, Italy; erika.montabone@unito.it (E.M.); renatocarigno@gmail.com (R.C.)

**Keywords:** rituximab, systemic sclerosis, scleroderma, intensified B-depletion therapy

## Abstract

Background: B-cells have been suggested to play a role in the pathogenesis of systemic sclerosis (SSc), representing, therefore, a potential therapeutic target. Objectives: We aimed at investigating the 36-month outcomes of 20 SSc patients who underwent an intensified B-depletion therapy (IBCDT) scheme, including both Rituximab (RTX) and cyclophosphamide (CYC). Methods: Data from 20 severe patients (18 females and 2 males, mean age 66.7 ± 11.0 years) with diffuse SSc (anti-topoisomerase I antibody in 95%) patients with multiorgan involvement including interstitial lung disease (ILD) treated with an IBCDT were prospectively collected. IBCDT comprehended: RTX 375 mg/m^2^ administered for four weekly doses (on days 1, 8, 15, and 22), followed by two additional doses after 30 and 60 days, in addition to two administrations of 10 mg/kg of intravenous CYC plus three methylprednisolone pulses (15 mg/kg) and subsequently followed by oral prednisone rapidly tapered to low minimum dosage of 5 mg daily. In addition, 10 patients with more severe functional respiratory impairment at baseline were also treated with RTX 500 mg every 4 months during the first year and two times a year during the second and the third year. Results: After 36 months of follow-up, we recorded significant amelioration in N-terminal-pro-brain natriuretic peptide (NT-proBNP) levels (mean 385.4 ± 517 pg/mL at baseline to 279 ± 543 after 36 months). In addition, a significant radiological improvement of ILD in 20% of patients (4/20) and a radiological stabilization with no sign of progression of interstitial involvement in 13/20 (65%) were documented. A total of 3 out of 20 (15%) patients experienced a worsening of the ILD. No patient showed further decrease in functional respiratory parameters, including forced vital capacity, forced expiratory volume in one second, and mean values of diffusing capacity for carbon monoxide Moreover, no patient showed any change in the ejection fraction and pulmonary artery pressure when comparing values at baseline and after 24 and 36 months of observation. No severe infection, renal flare, RTX-related side effects were observed. No patient died. Conclusions: Our findings support that the IBCDT was well tolerated and might be a promising therapeutic option for the management of SSc, especially in those subjects with multiorgan involvement that includes ILD.

## 1. Introduction

Systemic sclerosis (SSc) is characterized by the higher standardized mortality rate among systemic rheumatic diseases [1]. Several factors, including endothelial dysfunction and ad e regulation in fibroblast activation have been investigated to elucidate the pathogenesis of the diseases [2,3]. Nowadays, the mortality rate of severe forms of SSc is as high as 13% over a median follow-up of 3.0 years [4]. In this context, interstitial lung disease (ILD) represents the major cause of mortality. The management of severe SSc have mainly been centered on conventional immunosuppressant agents, such as cyclophosphamide (CYC) or mycophenolate [5,6,7,8]. Those therapeutic strategies, albeit associated with some degrees of efficacy, were not free from side effects, potentially limiting their use. The role of B-cell depletion therapy and hematopoietic stem cell transplantation have been intensively evaluated in the last decade for the management of severe cases of SSc, with some promising results [9,10]. A growing body of evidence coming from both animal and human research suggested that B-cells are able to promote and enhance the fibrotic process which represent the main pathogenic mechanisms in SSc development, making the use of B-cell depletion therapy in SS can appealing option [11]. Preliminary data on the use of Rituximab (RTX) in the management of SSc has shown promising results [12]. RTX is a chimeric mouse and human monoclonal antibody with binding specificity to CD20, a transmembrane protein expressed on B-lymphocytes from the pre-B-cell stage of development.

The rationale of targeting B-cells in patients with SSc relies on available data supporting that these cells might have a pathogenic role in this debilitating disease [2,3,9]. Chronic hyperactivity and amplified calcium responses after B-cell receptor (BCR) cross-linking have been shown in animal models of SSc (the so-called tight skin mice). Similarly, B-cells from mice models of SSc showed an activated profile as demonstrated by an increased expression of CD19 signaling (a crucial positive BCR response regulator) and impaired function of CD22 (a negative BCR response regulator). In line with these observations, an overexpression of CD19 has been observed in B-cells from patients with SSc, compared with B-cells from healthy donors and disease control patients. B-cells presented with a pro-inflammatory chronic profile [2,3]. Additionally, a growing body of evidence is supporting that B-cell genes can be specifically transcribed in SSc skin and that B-cell infiltration plays a critical pathogenic role in SSc-associated ILD [2,3]. Its use has been associated with an improvement of lung parameters and skin involvement. 

Indeed, the amelioration of the lung profile, as evaluated by the pulmonary function tests (PFTs), has been considered as an encouraging result in further exploring the use of RTX in SSc, as the worsening of PFTs over the years is a common clinical scenario for the affected subjects [13]. A large multicenter trial, led by the European League against Rheumatism Scleroderma Trial and Research group, have confirmed the potential efficacy targeting B-cells in order to improve SSc-related ILD [14,15].

We previously investigated the promising outcome of patients with severe autoimmune conditions, namely severe systemic lupus erythematosus, anti-neutrophil cytoplasmic antibodies (ANCA)-associated vasculitis, and cryoglobulinemic vasculitis, treated with an intensified B-cell depletion therapy (IBCDT)protocol, including RTX, CYC, and methylprednisolone pulses [16,17,18,19]. Safety and efficacy of the IBCDT scheme have been already proven both for the induction of remission and for long-term remission, despite the absence of further immunosuppressive maintenance therapies.

## 2. Aim

The aim of this study was to prospectively assess both safety and efficacy of the IBCDT protocol in a cohort of SSc patients with severe ILD.

## 3. Patients and Methods

We selected consecutive patients fulfilling the American College of Rheumatology criteria for diffuse SSc diagnosis, in association to ILD plus at least two of the following features: (a) pulmonary artery hypertension (PAH); (b) skin thickening; (c) esophagus involvement; (d) polyarthritis.

### 3.1. Protocol

#### Therapeutic Schedules

IBCDT: A total of 20 patients was treated with IBCDT, accordingly as previously described [16,17,18,19] to the following scheme: RTX 375 mg/m^2^ administered for four weekly doses (on days 1, 8, 15, and 22), followed by two more doses after 30 and 60 days, respectively, in addition to two administrations of 10 mg/kg of intravenous CYC (reduced, if necessary, according to renal impairment) at days 4 and 17, plus three methylprednisolone pulses (15 mg/kg) at days 1, 4, and 8, subsequently followed by oral prednisone (0.8 mg/kg daily) rapidly tapered to low dosage of 5 mg daily.

In addition, 10 patients (50%) who presented with a more severe functional respiratory impairment at baseline [defined as a diffusing capacity for carbon monoxide (DLCO) lower than 50%] were also treated with RTX 500 mg every 4 months during the first year and two times a year during the second and the third year.

This study was conducted according to the Piedmont and Aosta Valley (North West Italy) legislation for Rare Diseases (N. 1577/UC/SAN of 11.10.2005 based on Regional Government Act 23 April 2007 dealing with Rare Diseases, Systemic Sclerosis RM0091; article. 1: 796 paragraph Z Law number 296 of 2006. Number 5-5740).

The study was conducted according to the Helsinki Declaration and each patient provided written consent to participate.

### 3.2. Statistical Analysis

For the comparison of variables at baseline and follow-up, Student’s *t*-test was used for normally distributed parameters and the non-parametric Mann–Whitney test for non-normally distributed parameters. Correlations were calculated and significance was determined by Fisher’s exact test. Multivariable logistic regression analysis was used to identify any independent predictors of flare. Kaplan–Meier hazard plots were constructed for time to renal flare. For these analyses, the SPSS (IBM Corporation, Armonk, NY, USA) software was used. *p* < 0.05 was considered statistically significant.

### 3.3. Outcomes

#### Lung and Skin Fibrosis Assessment

Standard PFTs were assessed in all patients at baseline and then every 6 months during the 36 months of observation. PFTs evaluation included forced expiratory volume in one second (FEV1), forced vital capacity (FVC), and DLCO corrected for hemoglobin concentration. PFTs estimation were provided as a % of normal predicted values adjusted for sex, age, and height.

The diagnosis of PAH was assessed by echocardiography in all patients [20] and confirmed when indicated using right heart catheterization. High-resolution computed tomography (CT) scan findings were assessed during the follow-up as previously described [21].

The skin thickening evaluation was assessed by the Modified Rodnan Skin Score (mRSS) at baseline and every 6 months.

## 4. Results

A total of 20 patients with SSc (mean age 66.7 ± 11.0 years), including 18 female and 2 males, were enrolled in this study. The recruited patients presented with a severe form of diffuse SSc, involving different organs and systems, as follow: ILD was reported in 100% (20 out of 20) of the recruited patients, while PAH was present in 60% (12 out of 20) of the cohort, skin thickening in 85% (17 out of 20), esophagus involvement in 45% (9 out of 20), and polyarthritis in 25% (5 out of 20). Baseline characteristics are shown in Table 1.

**Figure 1:**
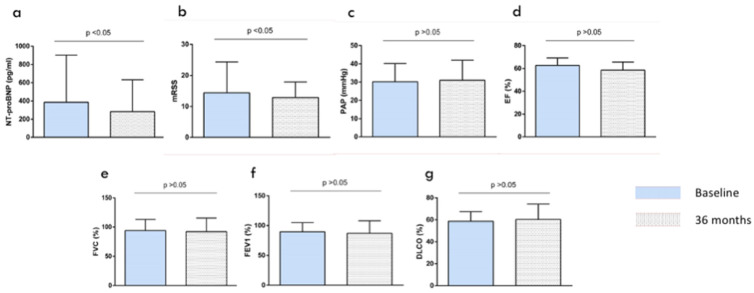
Patients characteristics at baseline and after 36 months of follow-up. Results are presented as mean ± standard error of the mean. (**a**) reduction in N-terminal-pro-brain natriuretic peptide (NT-proBNP) levels, (**b**) an improvement of the skin involvement as assessed by the Modified Rodnan Skin Score (mRSS) was documented, (**c**) no statistical significant changes in pulmonary artery pressure when comparing baseline and 36 month levels, (**d**) no patient showed any change in the ejection fraction, (**e**) forced vital capacity (FVC), (**f**) forced expiratory volume in one second (FEV1), (**g**) mean values of diffusing capacity for carbon monoxide (DLCO).

After a follow-up of 36 months, a reduction in N-terminal-pro-brain natriuretic peptide (NT-proBNP) levels (385.4 ± 517 pg/mL 283 ± 648 pg/mL, 279 ± 543 pg/mL, mean at baseline and after 24 and 36 months, respectively, *p* < 0.05) was observed (Figure 1a). Similarly, an improvement of the skin involvement as assessed by the mRSS was documented (14.4 ± 10.5 and 11.9 ± 9, comparing means at baseline and 36 months follow up, *p* < 0.05) (Figure 1b). In addition, after IBCDT treatment, in 20% of patients (4 out of 20) a significant radiological improvement of ILD evaluated by CT was documented. Moreover, in 13 out of 20 (65%) patients, radiological stabilization with no sign of progression of interstitial involvement has been reported. A total of 3 out of 20 (15%) patients experienced a worsening of the ILD.

No patient showed further decrease in PFT parameters, including FVC (93.6 ± 19.3, 92.2 ± 23.3, 91.2 ± 22.7, mean at baseline and after 24 and 36 months of observation, respectively), FEV1 (89.5 ± 15.6, 87 ± 21.2, 88 ± 23.1, mean at baseline and after 24 and 36 months, respectively), and mean values of DLCO (58.8 ± 8.6, 60.3 ± 14, 59.9 ± 13.2, at baseline and after 24 and 36 months) (Figure 1e–g). Moreover, no patient showed any change in the ejection fraction (mean baseline values: 62.8 ± 6.4, mean values at 24 months: 58.6 ± 7.1, mean values at 36 months: 59.5 ± 7.9) (Figure 1d) and in pulmonary artery pressure when comparing values at baseline and after 24 and 36 months of observation after IBCDT treatment (mean baseline: 30.2 ± 10.5, mean at 24 months: 31.1 ± 11.05, mean at 36 months: 32.2 ± 12.1) (Figure 1c). Changes in selected outcomes over time, at baseline and after 36 months of observation, are displayed in Figure 1 and Table 2.

A total of 10 patients (50%) were given a maintenance therapy of RTX 500 mg every 4 months during the first year and every 6 months during the second and third year, based on the more severe pulmonary involvement, defined as DLCO lower than 50% at baseline. None of them experienced a deterioration of the ILD.

No severe infection (e.g., leading to hospitalization or use of intravenous therapy) were observed during the follow-up. In detail, patients were strictly monitored for RTX-related side effects. No cases of serum sickness, nor late-onset neutropenia were recorded during the observation time of this study. No renal flare or deterioration of kidney function was observed. No patient died.

**Figure 2:**
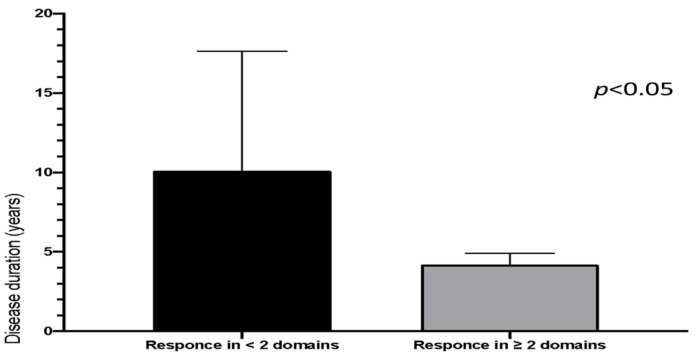
Response to intensified B-depletion therapy (IBCDT) related to systemic sclerosis (SSc) disease duration. Patients are divided in two groups according to the overall response to IBCDT in at least 2 SSc-domains (Modified Rodnan Skin Score, high resolution computed tomography lung, pulmonary function tests, N-terminal-pro-brain natriuretic peptide). Mean disease duration is significantly shorter in those patients who experienced a response to IBCDT in two or more SSc-domains (mean disease duration 4.2 years vs. 10.125 years). Y axis: Years, years of diseases duration (from diagnosis). Results are presented as mean ± standard error of the mean (SEM).

When separating patients for outcome, we observed that patients with shorter disease duration showed a higher prevalence of response in two or more SSc-domains (mRSS, high resolution CT lung, pulmonary function tests, NT-proBNP) (Figure 2). Indeed, patients with less than 5 years of disease duration were more likely to have an improvement in more than 2 items when compared to patients with longer disease duration (6 out of 9 vs. 2 out of 11, respectively, *p* = 0.0276).

When separating patients for outcome, no significant difference was observed when comparing patients in terms of age, antibodies profile, sex, or organ involvement.

When evaluated with a visual analog score (VAS, 0–10), patients perceived impact of the diseases on daily activities moved from mean 8 ± 2 to 4 ± 2 at the end of follow-up.

When applying multivariable logistic regression analysis to identify any independent predictors of flare, we did not find any robust candidate, probably due to the limited sample size.

## 5. Discussion

PAH and ILD represent the major causes of mortality among SSc patients. While we have experienced a marked improvement in the management of SSc, therapeutic strategies for some severe organ involvement such as ILD, are still suboptimal and with limited efficacy.

Over the last years, targeting B-cell has been investigated based on its promising role in SSc treatment, including lung and skin involvement. Indeed, the available data are suggesting B-cells to be crucial regulators of the evolution toward fibrotic processes [22].

Data from in vitro and animal models provided the rational for the use of B-cell depletion therapy in patients with SSc. Among others, it is worth mentioning some aspects. First, CD19 signaling is amplified in B-cell subpopulation as expressed by B-cell receptor response regulator in animal models of SSc [23] and those data are confirmed in human experiments [24]. Secondly, B-cells regulate fibroblasts in inducing an increase incollagen production via a transforming growth factor-β-dependent pathway [25], supporting the pathogenic mechanism through which B-cell depletion therapy can induce an improvement of skin fibrosis [26]. It is also important to remember that the skin biopsy of SSc patients show a B-cell signature [27]. Similarly, B-cell infiltration has been observed in biopsies from patients with SSc-associated ILD [28].

The above-mentioned observations have been confirmed in preliminary clinical experiences, including our own [29]. Our experience data further evidence supporting the utility of B-cell depletion strategies in the management of SSc.

It is usually difficult to evaluate the possible clinical benefit of a given therapeutic strategy in patients with SSc due to the intrinsic characteristics of the disease, including slowly progression, multiorgan involvement, heterogeneity of clinical outcome. Moreover, skin thickening can improve in late stages of the SSc, further increasing the variability in the assessment of the clinical outcomes. These are just some of the reasons why clinical studies with long-term follow-up are still warranted to obtain more definite answers about the potential benefit of any therapeutic strategies.

Our data support previous studies reporting that B-cell depletion positively impacts lung and skin outcomes in patients with SSc [30,31,32,33,34]. Interestingly, if compared to other available studies, our experience is supported by a longer-term follow-up. Only short to medium term data investigating the clinical benefit of RTX in SSc are currently available. None of them refers to the use of RTX combined with other immunosuppressants. The duration of follow-up of the available studies is crucial and still represent a limitation when evidence-based guidance is needed, for instance, the duration of treatment for SSc-associated ILD is still elusively addressed. To date, continuous treatment is recommended. This conclusion was clearly supported by the observation that CYC discontinuation after one year has been followed by a worsening in PFTs continued over the second-year follow-up [6]. Based on these observations some Authors speculated about the need of treatment administration on a long-term basis, regardless the fact that CYC-toxicity strongly limited its undefined uses. RTX could represent a promising therapeutic tool for SSc-related ILD, also because it can be safely administered for long on the basis of data derived from rheumatoid arthritis. Daoussis et al. [12] reported that all patients who stopped RXT deteriorated. Consequently, they concluded that patients who experienced favorable response to RTX should receive long-term maintenance. In line with these observations, we showed no progression of ILD in the 10 patients with DLCO lower than 50% at baseline who were treated per protocol with a RTX-based maintenance regimen. This observation is in line with the rational of the IBCDT: adding CYC to Rituximab does prolong B-cell depletion for the synergic effect of CYC on B assuring a longer-lasting remission [18,21]. The addition of CYC at relatively low dose (that limited CYC specific side effects) was mainly aimed at potentiating the B-cell depletion.

In the present study we also challenge the paradigm that steroid administration can inexorably induce a rapid deterioration of renal function. It is worth noticing that all data supporting this axiom are based on uncontrolled retrospective non-updated data exploring patients’ outcomes following any kind of therapy [35].

Finally, the time of IBDCT administration should be discussed, as we observed that patients with shorter diseases duration were more prone to have a response in more disease-related items. The topic has been recently reviewed by Konstantinos Melissaropoulos and co-workers [36]. In a case-based review they found that there may be a window of opportunity in SSc and highlights the need for clinical trials targeting very early or early disease. In detail, they reported on two cases where RTX was administered within 24 months from the appearance of Raynaud’s. In the first case, there was an improvement in interstitial lung disease as indicated by the improvement in pulmonary function tests and the regression of changes in high-resolution chest computed tomography (CT). In the second case, a good clinical response in skin fibrosis was observed.

None of the patients who received the extra RTX doses experienced a deterioration of the ILD.

However, no significant difference has been noted when comparing the patients who received the extra RTX doses to those who did not. The sample size and some degree of heterogeneity in the DLCO at baseline in the included cohort make further studies mandatory.

We acknowledge that our study, even though prospective, has the limitation not to be controlled. This may have led to some bias, and we are aware that conclusive answers should be supported from properly statistically powered randomized controlled trial. Similarly, the net benefit of our protocol needs to be confirmed in a controlled fashion in properly designed randomized controlled trials. Nevertheless, patients’ extended follow-ups are strengths for this study. Besides, while a physiological decline in PFTs can be observed in healthy individuals [37], the natural history of pulmonary function in patients with SSc-ILD is characterized in progressive fibrosing disease with a decline in FVC or DLCO values over time, an increase in lung high resolution CT extension, and a worsening of symptoms, leading to a high mortality [38,39].

## 6. Conclusions

Regardless of the progress in the management of SSc, some major manifestations, such as ILD, still significantly impact on morbidity and mortality of subjects with this condition. Our IBCDT protocol could represent a useful therapeutic approach in SSc setting, leading to control of SSc progression, especially in those patients who presented with severe lung and skin involvement, making superfluous in most cases a long-term administration of CYC or other conventional immunosuppressants. A properly designed randomized controlled trial is warranted to confirm our observations.

## Figures and Tables

**Table 1:** Baseline characteristics.

	*n*	%
Female	18	90
Age (Mean ± SD, Years)	66.7 ± 11.0	
Less than 5 Years Disease Duration	9	45
Raynaud’s	20	100
ILD	20	100
PAH	12	60
Skin Thickening	17	85
Esophagus Involvement	9	45
Polyarthritis	5	25
ANA	20	100
Anti-Topoisomerase I Antibodies (Scl-70)	19	95%
NT-proBNPLevels (Baseline, Mean ± SD, pg/mL)	385.4 ± 517	
mRSS (Baseline, Mean ± SD)	14.4 ± 10.5	
FVC (Baseline, Mean ± SD)	93.6 ± 19.3	
FEV1 (Baseline, Mean ± SD)	89.5 ± 15.6	
DLCO (B, Mean ± SD)	58.8 ± 8.6	
Ejection Fraction (Baseline, Mean ± SD)	62.8 ± 6.4	
Pulmonary Artery Pressure (Baseline, Mean ± SD)	30.2 ± 10.5	

SD, standard deviation; ILD, interstitial lung disease; PAH, pulmonary artery hypertension; mRSS, Modified Rodnan Skin Score; NT-proBNP, N-terminal-pro-brain natriuretic peptide, FVC, forced vital capacity, FEV1, forced expiratory volume in one second; DLCO, diffusing capacity for carbon monoxide. Clinical and Laboratory features (ILD, PAH, skin thickening, esophagus involvement, polyarthritis, ANA, anti-topoisomerase I antibodies) are reported as dichotomous variables (yes or no).

**Table 2:** Clinical profile during the follow-up.

	Baseline	24 Months	*p*-Value *	36 Months	*p*-Value **
Rodnan Skin Score	14.4 ± 10.5	12.9 ± 10	*p* = 0.073	11.9 ± 9	*p* < 0.05
NT-proBNP (mean ± SD, pg/mL)	385.4 ± 517	283 ± 648	*p* < 0.05	279 ± 543	*p* < 0.05
FVC (mean ± SD)	93.6 ± 19.3	92.2 ± 23.3	*p* = 0.77	91.2 ± 22.7	*p* = 0.63
FEV1 (mean ± SD)	89.5 ± 15.6	87 ± 21.2	*p* = 0.59	88 ± 23.1	*p* = 0.57
DLCO	58.8 ± 8.6	60.3 ± 14	*p* = 0.71	59.9 ± 13.2	*p* = 0.77
Ejection fraction (mean ± SD)	62.8 ± 6.4	58.6 ± 7.1	*p* = 0.45	59.5 ± 7.9	*p* = 0.34
pulmonary artery pressure (mean ± SD)	30.2 ± 10.5	31.1 ± 11.05	*p* = 0.51	32.2 ± 12.1	*p* = 0.49
eGFR (mL/min/1.73 m^2^)	71.4 ± 9.7	72.3 ± 10.6	*p* = 0.75	70.2 ± 11.3	*p* = 0.71

* Comparing baseline and 24-month follow-up; ** comparing baseline and 36-month follow-up; Student’s *t*-test was used for normally distributed parameters and the non-parametric Mann–Whitney test for non-normally distributed parameters. *p* < 0.05 was considered statistically significant.NT-proBNP, N-terminal-pro-brain natriuretic peptide; FVC, forced vital capacity, FEV1, forced expiratory volume in one second; DLCO, diffusing capacity for carbon monoxide; eGFR, Estimated glomerular filtration rate.

## Data Availability

Data will be available upon request to the corresponding author.

## References

[1] Elhai M., Meune C., Avouac J., Kahan Y. (2012). Allanore Trends in mortality in patients with systemic sclerosis over 40 years: A systematic review and meta-analysis of cohort studies. Rheumatology.

[2] Sakkas L., Chikanza I.C., Platsoucas C.D. (2006). Mechanisms of Disease: The role of immune cells in the pathogenesis of systemic sclerosis. Nat. Clin. Pract. Rheumatol..

[3] Denton C.P., Khanna D. (2017). Systemic sclerosis. Lancet.

[4] Hao Y., Hudson M., Baron M., Carreira P., Stevens W., Rabusa C., Tatibouet S., Carmona L., Joven B.E., Huq M. (2017). Early mortality in a multinational systemic sclerosis inception cohort. Arthritis Rheumatol..

[5] Tashkin D.P., Elashoff R., Clements P.J., Goldin J., Roth M.D., Furst D.E., Arriola E., Silver R., Strange C., Bolster M. (2006). Cyclophosphamide versus placebo in scleroderma lung disease. N. Engl. J. Med..

[6] Tashkin D.P., Elashoff R., Clements P.J., Roth M.D., Furst D.E., Silver R.M., Goldin J., Arriola E., Strange C., Bolster M.B. (2007). Effects of 1-year treatment with cyclophosphamide on outcomes at 2 years in scleroderma lung disease. Am. J. Respir. Crit. Care Med..

[7] Liossis S.N., Bounas A., Andonopoulos A.P. (2006). Mycophenolate mofetil as first-line treatment improves clinically evident early scleroderma lung disease. Rheumatology.

[8] Nihtyanova S.I., Brough G.M., Black C.M., Denton C.P. (2007). Mycophenolate mofetil in diffuse cutaneous systemic sclerosis—A retrospective analysis. Rheumatology.

[9] Smith V., Van Praet J.T., Vandooren B., Van Der Cruyssen B., Naeyaert J.-M., Decuman S., Elewaut D., De Keyser F. (2008). Rituximab in diffuse cutaneous systemic sclerosis: An open-label clinical and histopathological study. Ann. Rheum. Dis..

[10] Smith V., Piette Y., Van Praet J.T., Decuman S., Deschepper E., Elewaut D., De Keyser F. (2012). Two-year results of an open pilot study of a 2-treatment course with rituximab in patients with early systemic sclerosis with diffuse skin involvement. J. Rheumatol..

[11] Volkmann E.R., Varga J. (2019). Emerging targets of disease-modifying therapy for systemic sclerosis. Nat. Rev. Rheumatol..

[12] Daoussis D., Melissaropoulos K., Sakellaropoulos G., Antonopoulos I., Markatseli T.E., Simopoulou T., Georgiou P., Andonopoulos A.P., Drosos A., Sakkas L. (2017). A multicenter, open-label, comparative study of B-cell depletion therapy with Rituximab for systemic sclerosis-associated interstitial lung disease. Semin. Arthritis Rheum..

[13] Steen V.D., Conte C., Owens G.R., Medsger T.A. (1994). Severe restrictive lung disease in systemic sclerosis. Arthritis Rheum..

[14] Keir G.J., Maher T.M., Ming D., Abdullah R., De Lauretis A., Wickremasinghe M., Nicholson A.G., Hansell D.M., Wells A.U., Renzoni E.A. (2014). Rituximab in severe, treatment-refractory interstitial lung disease. Respirology.

[15] Jordan S., Distler J.H.W., Maurer B., Huscher D., Van Laar J.M., Allanore Y., Distler O. (2014). Effects and safety of rituximab in systemic sclerosis: An analysis from the European scleroderma trial and research (EUSTAR) group. Ann. Rheum. Dis..

[16] Roccatello D. (2017). “How I treat” autoimmune diseases: State of the art on themanagement of rare rheumatic diseases and ANCA-associated systemic idiopathicvasculitis. Autoimmun. Rev..

[17] Roccatello D., Sciascia S., Baldovino S., Rossi D., Alpa M., Naretto C., Di Simone D., Menegatti E. (2016). Improved (4 plus 2) Rituximab protocol for severe cases of mixed cryoglobulinemia: A 6-year observational study. Am. J. Nephrol..

[18] Roccatello D., Sciascia S., Di Simone D., Solfietti L., Naretto C., Fenoglio R., Baldovino S., Menegatti E. (2016). New insights into immune mechanisms underlying response to Rituximab in patients with membranous nephropathy: A prospective study and a review of the literature. Autoimmun. Rev..

[19] Roccatello D., Sciascia S., Baldovino S., Rossi D., Alpa M., Naretto C., Di Simone D., Simoncini M., Menegatti E. (2015). A 4-year observation in lupus nephritis patients treated with an intensified B-lymphocyte depletion without immunosoppressive maintenance treatment—Clinical response compared to literature and immunological re-assessment. Autoimmun. Rev..

[20] Ciurzyński M., Bienias P., Ciesielska K., Chrzanowska A., Dudzik-Niewiadomska I., Kurnicka K., Domienik-Karłowicz J., Siwicka M., Sobieraj P., Kalińska-Bienias A. (2019). Accuracy of Doppler echocardiography in the hemo-dynamic assessment of pulmonary circulation in patients with systemic sclerosis. Adv. Med. Sci..

[21] Goldin J.G., Lynch D.A., Strollo D.C., Suh R.D., Schraufnagel D.E., Clements P.J., Elashoff R.M., Furst D.E., Vasunilashorn S., McNitt-Gray M.F. (2008). High-resolution CT scan findings in patients with symptomatic scleroderma-related interstitial lung disease. Chest.

[22] Mavropoulo A., Simopoulou T., Varna A., Liaskos C., Katsiari C.G., Bogdanos D.P., Sakkas L.I. (2016). Breg cells are numerically decreased and functionally impaired in patients with systemic sclerosis. Arthritis Rheumatol..

[23] Asano N., Fujimoto M., Yazawa N., Shirasawa S., Hasegawa M., Okochi H., Tamaki K., Tedder T.F., Sato S. (2004). B lymphocyte signaling established by the CD19/CD22 loop regulates autoimmunity in the tight-skin mouse. Am. J. Pathol..

[24] Sato S., Fujimoto M., Hasegawa M., Takehara K. (2004). Altered blood B lymphocyte homeostasis in systemic sclerosis: Expanded naive B cells and diminished but activated memory B cells. Arthritis Rheum..

[25] Francois A., Chatelus E., Wachsmann S., Sibilia J., Bahram S., Alsaleh G., Gottenberg J.-E. (2013). Blymphocytes and B-cell activating factor promote collagen and profibrotic markers expression by dermal fibroblasts in systemic sclerosis. Arthritis. Res. Ther..

[26] Hasegawa M., Hamaguchi Y., Yanaba K., Bouaziz J.D., Uchida J., Fujimoto M., Matsushita T., Matsushita Y., Horikawa M., Komura K. (2006). B-lymphocyte depletion reduces skin fibrosis and autoimmunity in the tight-skin mouse model for systemic sclerosis. Am. J. Pathol..

[27] Whitfield M.L., Finlay D.R., Murray J.I., Troyanskaya O.G., Chi J.T., Pergamenschikov A., McCalmont T.H., Brown P.O., Botstein D., Connolly M.K. (2003). Systemic and cell type-specific gene expression patterns in scleroderma skin. Proc. Natl. Acad. Sci. USA.

[28] Lafyatis R., O’Hara C., Feghali-Bostwick C.A., Matteson E. (2007). B cell infiltration in systemic sclerosis-associated interstitial lung disease. Arthritis Rheum..

[29] Rossi D., Cecchi I., Radin M., Rubini E., Sciascia S., Roccatello D. (2018). SAT0477 Intensified B-cell depletion therapy in progressive systemic sclerosis patients: 24 months follow-up. Ann. Rheum. Dis..

[30] Hughes M., Denton C.P., Khanna D. (2020). Rituximab for the treatment of systemic sclerosis-interstitial lung disease. Rheumatology.

[31] Goswami R.P., Ray A., Chatterjee M., Mukherjee A., Sircar G., Ghosh P. (2020). OUP accepted manuscript. Rheumatology.

[32] Zhu L., Chung M.P., Gagne L., Guo H.H., Guenther Z., Li S., Jacobs S., Morisset J., Mooney J.J., Raj R. (2020). Rituximab versus mycophenolate in the treatment of recalcitrant connective tissue disease–associated interstitial lung disease. ACR Open Rheumatol..

[33] Giuggioli D., Lumetti F., Colaci M., Fallahi P., Antonelli A., Ferri C. (2015). Rituximab in the treatment of patients with systemic sclerosis. Our experience and review of the literature. Autoimmun. Rev..

[34] Narváez J., Pirola J.P., LLuch J., Juarez P., Nolla J.M., Valenzuela A. (2019). Effectiveness and safety of rituximab for the treatment of refractory systemic sclerosis associated calcinosis: A case series and systematic review of the literature. Autoimmun. Rev..

[35] Daoussis D., Tsamandas A., Antonopoulos I., Filippopoulou A., Papachristou D.J., Papachristou N.I., Andonopoulos A.P., Liossis S.N. (2016). B cell depletion therapy upregulates Dkk-1 skin expression in patients with systemic sclerosis: Association with enhanced resolution of skin fibrosis. Arthritis Res. Ther..

[36] Trang G., Steele R., Baron M., Hudson M. (2010). Corticosteroids and the risk of scleroderma renal crisis: A systematic review. Rheumatol. Int..

[37] Melissaropoulos K., Kraniotis P., Bogdanos D.P., Dimitroulas T., Sakkas L., Daoussis D. (2019). Targeting very early systemic sclerosis: A case-based review. Rheumatol. Int..

[38] Alvis B.D., Hughes C.G. (2015). Physiology considerations in geriatric patients. Anesthesiol. Clin..

[39] Kolb M., Vašáková M. (2019). The natural history of progressive fibrosing interstitial lung diseases. Respir. Res..

